# Clinical and Genetic Characteristics of *Enterobacter cloacae* and *Klebsiella aerogenes* in Children

**DOI:** 10.3390/microorganisms14020292

**Published:** 2026-01-27

**Authors:** Ki Wook Yun, Ye Eun Kim, Dayun Kang, Hye Jeong Moon

**Affiliations:** 1 Department of Pediatrics, College of Medicine, Seoul National University, Seoul 03080, Republic of Korea; a01068863058@gmail.com; 2 Department of Pediatrics, Seoul National University Children’s Hospital, Seoul 03080, Republic of Korea; dayun0333@gmail.com (D.K.); mhj8027@naver.com (H.J.M.)

**Keywords:** *Enterobacter cloacae*, *Klebsiella aerogenes*, bloodstream infection, whole-genome sequencing, children

## Abstract

This study investigated the clinical and genomic characteristics of *Enterobacter cloacae* complex (ECC) and *Klebsiella aerogenes* bloodstream infections (BSIs) in pediatric patients. A total of 115 BSI episodes (ECC: 86, *K. aerogenes*: 29) from 110 children hospitalized between 2011 and 2024 were retrospectively analyzed. Whole-genome sequencing was performed on available isolates to determine species, sequence types, and antimicrobial resistance (AMR) genes. Clinical characteristics, antibiotic usage, and outcomes were compared between groups. Patients with *K. aerogenes* BSI were younger and more likely to be preterm or diagnosed with urosepsis, while ECC infections were more frequently associated with hematologic malignancies. According to a multivariable analysis of the entire cohort (*n* = 115), *K. aerogenes* infection (OR [6.26], 95% CI [1.36–28.78]) and gentamicin resistance (OR [10.06], 95% CI [1.88–53.87]) were independently associated with 30-day mortality. *Enterobacter hormaechei* was the most common ECC species (68.4%) and exhibited the highest prevalence of AMR genes, particularly those conferring resistance to aminoglycosides, β-lactams, and trimethoprim–sulfamethoxazole. In contrast, *K. aerogenes* harbored few resistance genes. Multi-locus sequence typing analysis revealed high genetic diversity in both ECC and *K. aerogenes*, without evidence of dominant clonal expansion. Despite similarities in clinical presentation, ECC and *K. aerogenes* exhibit distinct age distributions, resistance profiles, and genetic diversity in pediatric BSIs. These findings underscore the importance of species-level identification and continued genomic surveillance to inform empirical antibiotic strategies and prevent the spread of resistant strains.

## 1. Introduction

Antibiotic-resistant Gram-negative bacteria (GNBs) have emerged as a significant global health concern, affecting the long-term prognosis of patients with various chronic diseases. In the late 1990s and early 2000s, extended-spectrum β-lactamase (ESBL)-producing *Escherichia coli* and *Klebsiella pneumoniae* were the primary antibiotic-resistant GNBs of concern. Since then, carbapenem-resistant *Acinetobacter baumannii* and *Pseudomonas aeruginosa* have emerged as problematic. *Enterobacter* species, which inherently possess the β-lactamase AmpC, can develop inducible resistance to third-generation cephalosporins. Recently, certain *Enterobacter* strains have also been shown to be resistant to carbapenem, forming a significant proportion of carbapenem-resistant *Enterobacteriaceae* (CRE), with increasing detection rates in clinical settings [1,2,3,4].

In 2017, *Enterobacter aerogenes* were reclassified as *Klebsiella aerogenes* based on phylogenetic evidence [5]. The remaining members of the genus *Enterobacter* can be grouped into 22 distinct phylogenetic groups, which are together referred to as the *Enterobacter cloacae* complex (ECC). Differences in clinical risk factors, antibiotic susceptibility patterns, and patient outcomes between the ECC and *K. aerogenes* have been investigated [6,7,8,9], but the conclusions have been inconsistent; moreover, no studies have been conducted in children. A better understanding of the differences between these two main GNBs may allow us to develop more appropriate empirical antibiotic therapies and improve outcomes in children. Therefore, this study aimed to identify the clinical and genetic differences between the ECC and *K. aerogenes* isolated from blood in Korean children.

## 2. Materials and Methods

### 2.1. Data Collection and Definitions

All patients aged 20 years or younger with ECC or *K. aerogenes* bloodstream infection (BSI) who were admitted to Seoul National University Children’s Hospital (SNUCH) between January 2011 and December 2024 were identified using the clinical data warehouse (CDW). If patients had multiple episodes of ECC or *K. aerogenes* BSI, only the first episode was included. Patient demographic and clinical information—including underlying diseases, laboratory results, antibiotics use, and clinical outcomes—was collected retrospectively through the CDW and medical chart review. The blood culture samples were collected during routine patient clinical care and were transported to the microbiology unit of the laboratory. Bacterial species identification and antimicrobial susceptibility testing (AST) were performed using a VITEK2 automated system (BioMériux, Marcy l’Étoile, Lyon, France). The results were retrospectively collected and interpreted according to the Clinical and Laboratory Standards Institute (CLSI) guidelines [10].

In routine clinical care, organism identification from standard clinical microbiology methods is reported to the treating team prior to final AST results; WGS-based species identification was performed retrospectively for research and did not influence real-time treatment decisions. Central line-associated bloodstream infection (CLABSI) was defined as a laboratory-confirmed BSI in a patient with a central venous catheter in place, with no alternative primary infection source identified on clinical review that better explained the bacteremia, consistent with standard surveillance definitions. Recurrence was defined as a repeat episode of bacteremia due to the same organism occurring ≥30 days after the index episode with documented intervening blood culture negativity.

### 2.2. Whole-Genome Sequencing

We performed whole-genome sequencing (WGS) on 38 ECC (44.2%) and 9 *K. aerogenes* strains (31.0%) that were isolated from the study cases. Genomic DNA (gDNA) was prepared using the MasterPure™ Complete DNA Purification Kit (Lucigen, Middleton, WI, USA) according to the manufacturer’s instructions. The gDNA was qualitatively checked using a NanoDrop™ (Thermo Fisher Scientific, Waltham, MA, USA) and quantified using a 1× dsDNA HS Assay Kit on a Qubit™ 4 fluorometer (Thermo Fisher Scientific). Equal amounts of gDNA (~200 ng) from each isolate were used for library preparation using the Rapid Barcoding Sequencing Kit (SQK-RBK114.24) according to the manufacturer’s protocol (Oxford Nanopore Technologies, Oxford, UK). Pooled libraries of six to ten isolates were run on a FLO-MIN114 flow cell with MinION next-generation sequencing (NGS) equipment (MinION Mk1B, Oxford Nanopore Technologies) and MinKNOW software v.24.06.14 (Oxford Nanopore Technologies) for 36 h. Basecalling was performed in Guppy software with a high-accuracy model, v.4.3.0 (Oxford Nanopore Technologies). The base-called data were packed into FASTQ with a maximum of 4000 reads per file.

The median read N50 across the 47 isolates was 4761 K (IQR 4676–5044 K), and the mean depth of coverage was 136.7x (SD 100.0x)/median 98.5x (IQR 58.5–189.6x). These metrics supported downstream assembly and variant calling using the high-accuracy basecalling model. Detailed per-isolate sequencing metrics are provided in Supplementary Table S1.

### 2.3. Species Identification

ECC species identification was performed via the Taxonomic Profiling tool of CLC Genomics Workbench v.24.0.2 (Qiagen, Hilden, Germany). Reads were mapped to a reference genome database (GenBank and RefSeq assembly accessions) and were assigned to a reference genome or higher taxonomic level on the basis of their mapping quality score, i.e., the confidence that the read was correctly mapped. Following read mapping, qualification and quantification steps were performed to refine the results. Bacterial species were confirmed when the top-ranked species included >90% of the sequences that were tested.

### 2.4. Multilocus Sequence Typing

The sequences of the seven genes (*dnaA*, *fusA*, *gyrB*, *leuS*, *pyrG*, *rplB*, and *rpoB* for both *K. aerogenes* and the ECC) used for multilocus sequence typing (MLST) were automatically extracted from the WGS reads and compared with the reference allele sequences in the PubMLST database (https://pubmlst.org/organisms, accessed on 22 February 2025) via the CLC Genomics Workbench. The sequence type (ST) was determined on the basis of the individual schemes for ECC and *K. aerogenes* MLST. The relationships between the STs were visualized with all strains registered in the PubMLST database until 22 February 2025, by using the GrapeTree and ReporTree analysis tools on PubMLST.

### 2.5. Antimicrobial Resistance Gene Detection

Antimicrobial resistance (AMR) gene detection was performed using the Find Resistance with Nucleotide Database tool of the CLC Genomics Workbench. The tool uses the Basic Local Alignment Search Tool of the National Center for Biotechnology Information of the National Library of Medicine, USA, for the identification of acquired AMR genes in WGS data. The AMR Comprehensive Antibiotic Resistance Database (CARD, https://card.mcmaster.ca/, accessed on 22 February 2025) component aligns input reads with the minmap2 program against all of the reference sequences available in the CARD, which includes protein references, drug classes, and resistance ontologies that describe associated resistance mechanisms.

### 2.6. Statistical Analysis

We performed statistical analyses using SPSS version 29.0 software for Windows (IBM SPSS, Chicago, IL, USA). Statistical comparisons between groups for continuous variables were made with the Mann–Whitney U test or Student’s *t* test. For categorical variables, comparisons were made via the chi-square test or Fisher’s exact test. A *p* value < 0.05 was considered to indicate statistical significance.

## 3. Results

### 3.1. Subject Characteristics

During the study period, a total of 115 BSIs caused by the ECC (*n* = 86, 74.8%) or *K. aerogenes* (*n* = 29, 25.2%) were identified in 110 patients at SNUCH. The median age of the patients was 4.3 (interquartile range [IQR], 0.3–11.9) years, 47.0% of the patients were male, and all but 3 patients (97.3%) had underlying disease. The most common underlying condition was haemato-oncologic disease (HOD, 50.4%), followed by early prematurity (defined as <34 weeks of gestational age; 15.7%) and gastrointestinal/hepatobiliary disease (15.7%) (Table 1).

Among all the BSI cases, CLABSI accounted for 58.3%, and urosepsis accounted for 7.0%. The most commonly administered empirical 1st-generation antibiotics with GNB coverage were piperacillin–tazobactam (PIP-TAZ; *n* = 70, 60.9%), followed by meropenem (*n* = 32, 27.8%) and 3rd-generation cephalosporins (*n* = 7, 6.1%). However, among the patients who started with PIP-TAZ, 53 (75.7%) switched to meropenem before the AST results were obtained. As a result, 76.5% of patients received meropenem as an empirical antibiotic for ECC/*K. aerogenes* BSI. The overall in-hospital mortality was 16.5% (*n* = 19), and the attributable mortality for ECC/*K. aerogenes* BSI was 7.8% (*n* = 9). The all-cause 30-day and 100-day mortalities were 9.6% and 15.7%, respectively (Table 1).

### 3.2. Clinical and Microbiological Characteristics of ECC and K. aerogenes

**Figure 1:**
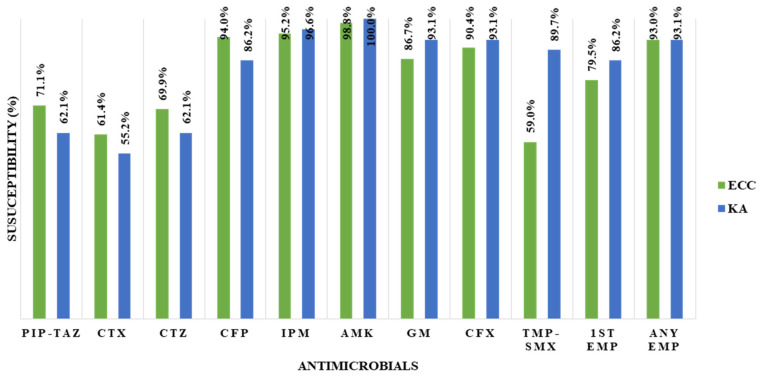
Comparison of antimicrobial susceptibility (%) between the *Enterobacter cloacae* complex and *Klebsiella aerogenes* groups. PIP-TAZ, piperacillin–tazobactam; CTX, cefotaxime; CTZ, ceftazidime; CFP, cefepime; IPM, imipenem; AMK, amikacin; GM, gentamycin; CFX, ciprofloxacin; TMP-SMX, trimethoprim–sulfamethoxazole; EMP, empirical antibiotics; ECC, *Enterobacter cloacae* complex; KA, *Klebsiella aerogenes.*.

Compared with patients with ECC infection, patients with *K. aerogenes* infection had a significantly lower median age (0.3 vs. 7.8 years, *p* = 0.001), higher proportions of early prematurity (<34 weeks of gestational age; 27.6% vs. 11.6%, *p* = 0.031), urosepsis (17.2% vs. 3.5%, *p* = 0.027), and 3rd-generation cephalosporin use as empirical antibiotics (17.2% vs. 2.3%, *p* = 0.012), and a higher all-cause 30-day mortality rate (20.7% vs. 5.8%, *p* = 0.022) (Table 1). On the other hand, patients with ECC infection had higher proportions of HOD (60.5% vs. 20.7%, *p* < 0.001) and meropenem use as an empirical antibiotic (81.4% vs. 62.1%, *p* = 0.029). There was no significant difference in the antimicrobial susceptibility rate between the ECC and *K. aerogenes*, except for trimethoprim–sulfamethoxazole (TMP-SMX; 59.0% and 89.7%, respectively; *p* = 0.003), or in antimicrobial susceptibility to empirical antibiotics (93.0% and 93.1%, respectively; *p* = 1.000) (Figure 1).

### 3.3. Species Identification and MLST of the ECC and K. aerogenes

Among the 38 ECC strains analysed, *E. hormaechei* was most frequently identified (*n* = 26, 68.4%), followed by *E. kobei* (*n* = 4, 10.3%), *E. asburiae* (*n* = 3, 7.7%), *E. ludwiggii* (*n* = 2, 5.1%), *E. roggenkampii* (*n* = 2, 5.1%), and *E. bugandensis* (*n* = 1, 2.6%). Among all the ECC strains, a total of 32 STs were identified, with ST90 and ST133 being the most common (*n* = 3 for each), followed by ST127 and ST527 (*n* = 2 for both). These four STs were identified exclusively in *E. hormaechei*. All other STs were exclusively identified in each strain. In conclusion, 26 *E. hormaechei* strains had 20 STs, and the other ECC species had the same number of STs as the strains included. For *K. aerogenes* (*n* = 9), six STs were identified: ST93 (*n* = 3, 33.3%), ST4 (*n* = 2, 22.2%), ST224 (*n* = 1, 11.1%), ST246 (*n* = 1, 11.1%), ST363 (*n* = 1, 11.1%), and ST389 (*n* = 1, 11.1%). Among these, ST93, ST4, and ST389 were assigned to the same clonal complex (*n* = 6, 66.7%).

### 3.4. Phylogenetic Tree of the ECC and K. aerogenes Based on MLST

**Figure 2:**
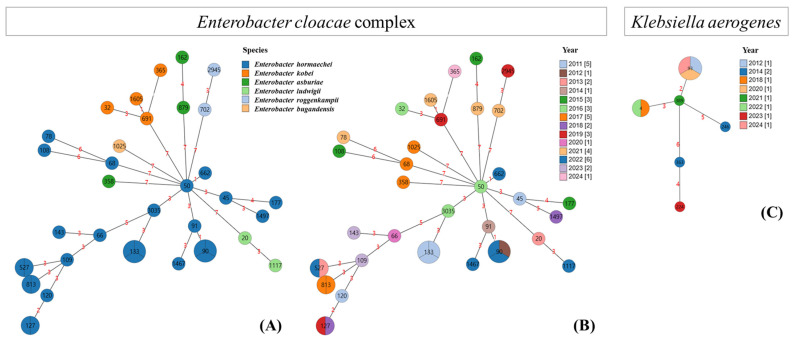
Phylogenetic tree with sequence types of the *Enterobacter cloacae* complex according to species (**A**) and year of isolation (**B**) and *Klebsiella aerogenes* according to year of isolation (**C**). Red numbers on the connecting lines indicate the number of allelic differences (i.e., differing MLST loci) between sequence types.

The ECC isolates in the present study were closely clustered by species in the phylogenetic tree, whereas the isolates of different species presented entirely distinct MLST profiles, with all seven alleles differing. Even within the same species, most isolates presented differences in at least three alleles, indicating a high degree of MLST heterogeneity (Figure 2A). Additionally, no clear associations between species or STs and the year of isolation were observed (Figure 2B). Similarly, *K. aerogenes* isolates presented significant differences in allele profiles among different STs, and no notable correlation with the year of isolation was identified (Figure 2C).

The size of the circles represents the number isolated. The number inside the circle in black indicates the sequence type (ST). The number in red on the line between circles indicates the number of alleles in the MLST genes that were different between the STs in the circles. The pie chart inside the circle indicates the number of bacterial isolates included in the corresponding STs

**Figure 3:**
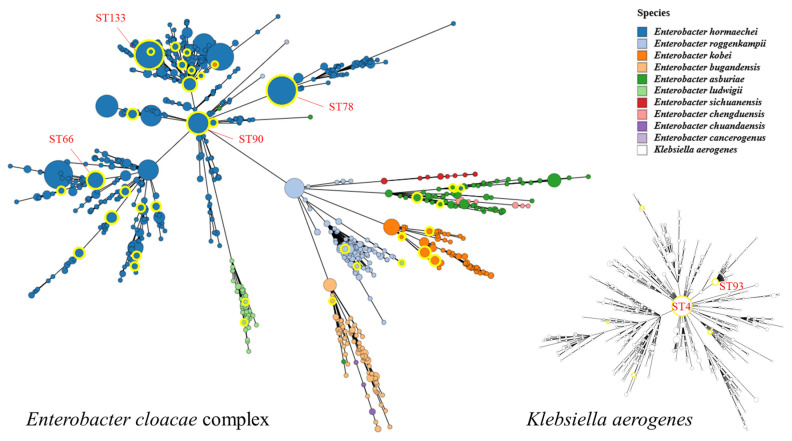
Phylogenetic tree with sequence types of all *Enterobacter cloacae* complex and *Klebsiella aerogenes* strains registered in the PubMLST database. The yellow circle indicates the isolate, including the strains isolated in the present study. *E. sichuanensis* and *E. chengduensis* reflect strains from the PubMLST reference database and were not observed among our study isolates.

ECC and *K. aerogenes* isolates registered in the PubMLST database as of 22 February 2025, were retrieved, including only those with fully assigned STs on the basis of complete allele information. In the case of the ECC, isolates lacking properly recorded species identification were excluded. As a result, a phylogenetic tree based on MLST was constructed, incorporating a total of 1542 ECC and 936 *K. aerogenes* isolates, including 39 newly registered ECC and 9 newly registered *K. aerogenes* isolates from this study (Figure 3). Similarly to previous findings, the ECC isolates were clearly distinguished by species via phylogenetic analysis, and the distribution of our isolates revealed considerable genetic distance within the tree. Additionally, ST90 and ST133, which had relatively high numbers of isolates in this study, were also the dominant STs in the global collection. A similar pattern was observed for *K. aerogenes*.

### 3.5. Antimicrobial Resistance Genes Detected in ECC and K. aerogenes

A total of 32 AMR genes were detected. Among them, AMR genes identified in at least three isolates were analysed across bacterial species, revealing that *E. hormaechei* harboured the highest number of AMR genes, whereas *K. aerogenes* had almost no AMR genes (Table 2). Aminoglycoside resistance genes, particularly *AAC*, *aadA*, and *ANT*, were more frequent in *E. hormaechei*. The *ACT* gene, encoding the β-lactamase AmpC, was present in all *E. hormaechei* isolates but absent in all *E. roggenkampii* (*n* = 2) and *K. aerogenes* (*n* = 9) isolates. However, both *E. roggenkampii* isolates harboured the *MIR* gene, another AMR gene encoding the β-lactamase AmpC. The *CTX*-*M*, *OXA*, and *TEM* genes were also more common in *E. hormaechei*, with *SHV* detected exclusively in this species. Resistance genes for TMP-SMX (*dfrA* and *sul*), quinolone (*Qnr*), colistin (*MCR*), and antiseptics (*qacE*) were also more prevalent in *E. hormaechei*.

**Figure 4:**
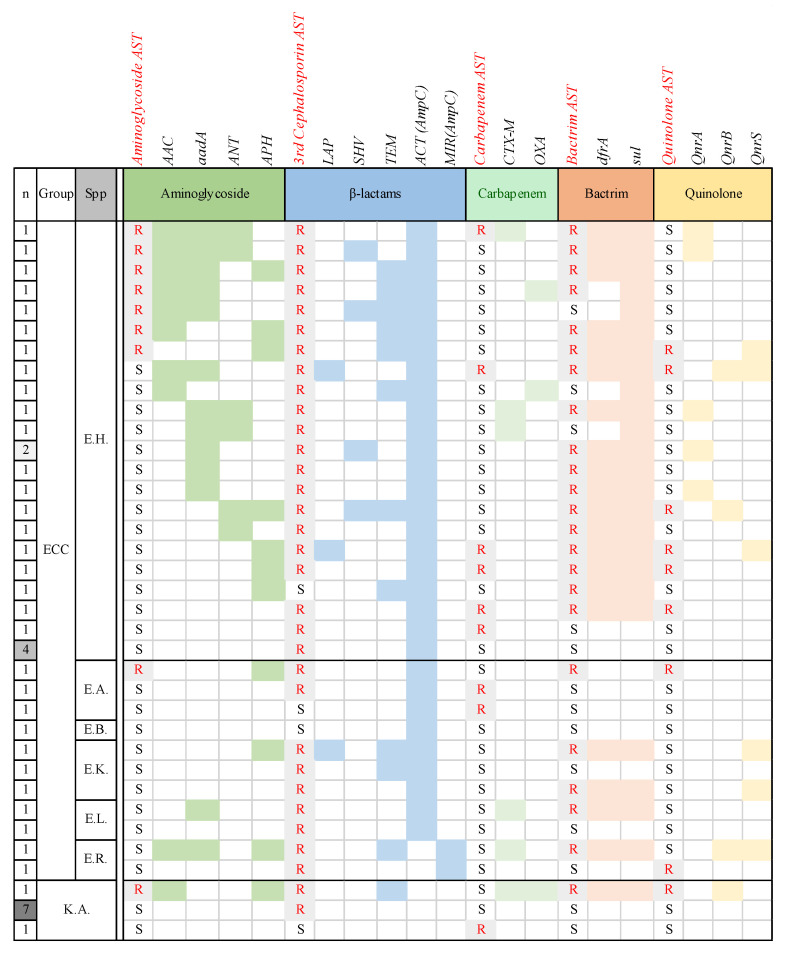
Comparison of the antibiotic resistome profiles of the *Enterobacter cloacae* complex and *Klebsiella aerogenes* strains isolated from children with bloodstream infection. AST, antimicrobial susceptibility testing; n, number; Spp, species; ECC, *E. cloacae* complex; E.H., *E. hormaechei*; E.A., *E. asburiae*; E.B., *E. bugandensis*; E.K., *E. kobei*; E.L., *E. ludwiggii*; E.R., *E. roggenkampii*; K.A., *K. aerogenes*; R, resistant; S, susceptible. Antimicrobial agents shown in red above the heatmap denote those for which phenotypic resistance (red “R”) was observed in at least one isolate.

A comparison of the AST results and AMR gene detection at the species and isolate levels revealed high strain diversity (Figure 4). The crude correlation between resistance phenotypes and AMR gene detection was shown for aminoglycosides (*AAC*), 3rd-generation cephalosporins (*ACT/MIR*), TMP-SMX (*dfrA*), and quinolones (*QnrB/S*). However, carbapenem resistance was not clearly associated with *CTX*-M or *OXA* gene detection.

## 4. Discussion

This study aimed to analyse the characteristics of *Enterobacter* spp. and *K. aerogenes*, which were recently diverged from each other but remain clinically and microbiologically similar. The findings revealed that *K. aerogenes* was more frequently associated with BSIs in preterm infants and patients with UTIs, whereas ECC was more commonly found in patients with HOD. The AMR genes were most frequently detected in *E. hormaechei*. Notably, genes conferring resistance to aminoglycosides were significantly more prevalent. In contrast, most *K. aerogenes* isolates did not harbour AMR genes. Among the ECC isolates, *E. hormaechei* accounted for the majority; however, neither *E. hormaechei* nor *K. aerogenes* represented a dominant clone.

BSIs caused by multidrug-resistant (MDR) Enterobacter species are critical contributors to mortality. Notably, the emergence of resistance has been observed in 19% of patients treated with third-generation cephalosporins [11,12]. In recent years, the emergence of ESBL- and/or carbapenemase-producing strains has further reinforced their importance as clinically relevant MDR pathogens [2,13]. A review of 434 GNB bacteraemia cases at SNUCH (1994–2001) identified Enterobacter spp. in 17.5%. Among the 72 Enterobacter isolates, 63.9% were extended-spectrum cephalosporin-resistant strains [14]. In the present study, 37.2% of the ECC isolates and 44.8% of the *K. aerogenes* isolates were resistant to third-generation cephalosporins, whereas carbapenem resistance was observed in 8.1% and 6.9%, respectively. Although the rate of resistance to third-generation cephalosporins was lower than that reported in previous studies, concerns remain regarding the potential for inducible resistance due to AmpC. Additionally, increasing carbapenem resistance rates highlight the need for close monitoring and careful antimicrobial stewardship.

Numerous studies in adults have compared the clinical characteristics of ECC and *K. aerogenes*, yielding various results. Most studies, consistent with the present findings, have reported higher AMR in the ECC [8,15] and a stronger association between *K. aerogenes* and UTIs [6,8,16]. However, the impact on patient outcomes has been inconsistent. For example, a U.S. study of 150 patients with *K. aerogenes* BSI found no significant differences in overall (28% vs. 21%) or attributable (20% vs. 12%) in-hospital mortality compared with those associated with ECC BSI. However, poor clinical outcomes, including mortality before discharge, recurrent BSI, and complications, were more common in patients with *K. aerogenes* BSI (70% vs. 40%) [15]. Another study analysing 172 *E. cloacae* and 67 *K. aerogenes* bacteraemia cases revealed that while *E. cloacae* exhibited higher AMR, *K. aerogenes* was more often linked to worse clinical outcomes, with overall mortality at discharge being 15.1% for *E. cloacae* and 17.9% for *K. aerogenes* [16]. Additionally, in the present study—the first conducted in a paediatric population—*K. aerogenes* was found to be associated with a higher mortality rate than ECC. However, in contrast, several adult studies have reported that ECC infection has either worse outcomes than *K. aerogenes* infection does or that there is no significant difference in outcomes between the two pathogens [6,8,9,17]. The variation in research findings is likely due to differences in study settings and the influence of various factors.

The higher 30-day mortality observed among children with *K. aerogenes* BSI may reflect, at least in part, host-related vulnerability: the *K. aerogenes* group comprised markedly younger patients with a higher proportion of prematurity, which may amplify the clinical impact of bacteremia. In addition, the independent association between gentamicin resistance and mortality suggests that early adequacy of empiric therapy could be particularly consequential in this subgroup. Although our genomic analyses primarily focused on AMR determinants and population structure, we cannot exclude the possibility that unmeasured virulence-related features contribute to outcome differences; this remains hypothesis-generating and warrants future studies incorporating systematic assessment of virulence-associated loci and host–pathogen interactions in pediatric BSIs.

Phenotype-based identification of Enterobacter species is often inaccurate, leading to an increasing number of studies utilizing WGS for more precise classification. Wu et al. [18] conducted a genomic study on 48 cases of Enterobacter-associated BSI using WGS to identify the exact species. The strains were classified into 12 species, with *Enterobacter xiangfangensis* (*n* = 21) being the most prevalent at a single centre in western China from 2016 to 2018 [18]. Similarly, Sarangi et al. [19] analysed the antimicrobial susceptibility profiles and genetic backgrounds of 42 ECC blood isolates collected from 42 hospitals across Japan between 2017 and 2019 using WGS. They reported that *E. hormaechei* was the most prevalent species (59.5%), followed by *E. roggenkampii* and *E. kobei* (both 11.9%) [19]. Other studies have also indicated that *E. hormaechei* is the most commonly detected ECC species in human infections and is strongly associated with high levels of antibiotic resistance [20,21]. Consistent with this, in the present study, species identification using WGS confirmed *E. hormaechei* as the most frequently detected species, with the highest number of strains registered in GenBank. These findings suggest that *E. hormaechei* is currently the most dominant Enterobacter species worldwide.

In recent years, MLST has become the most widely used method for analysing genotypic variations within the same species. Izdebski et al. [22] conducted a multinational study on the clonality of *E. cloacae* across Europe from 2008 to 2011 using the MLST scheme. Among the 173 ECC isolates resistant to expanded-spectrum cephalosporins, 88 STs were. The most widespread STs were ST66, ST78, ST108, and ST114 [22]. In another study, Emeraud et al. [20] genetically characterized 149 *VIM*-producing ECC isolates collected in France from 2015 to 2018. The major STs identified were ST873 (17.5%) and ST66 (12.1%). They concluded that the spread of *VIM*-producing ECC isolates is not driven by a single dominant clone due to their genetic diversity [20]. In addition, studies conducted in Japan and China revealed significant genetic diversity among ECC isolates, with no dominant STs [19,21]. Additionally, in the present study, MLST types were highly diverse, so the most frequently detected types (ST133 and ST90) each were identified in only three *E. hormaechei* isolates, accounting for only 7.9% of the total. *K. aerogenes* also presented a highly diverse range of STs. Compared with the distribution of STs registered in the global database, relatively dominant STs were indeed detected in this study. However, a wide variety of STs were also sporadically identified, indicating substantial genetic diversity within the isolates. These findings suggest that ECC exhibits not only species diversity but also considerable genotypic diversity. Rather than the expansion of a single dominant clone, various ECC strains persist in human and environmental reservoirs, sporadically causing opportunistic infections. This speculation might similarly be applied to *K. aerogenes*.

AmpC enzymes are chromosomal and inducible in most Enterobacter species. While AmpC-inducible species may initially appear susceptible to third-generation cephalosporins, the clinical use of these antibiotics can lead to the selection of resistant mutants, resulting in treatment failure [23,24]. Therefore, cefepime or carbapenems remain the preferred treatment, as recommended by IDSA guidelines [25]. In the present study, all the Enterobacter isolates carried AmpC. Except for *E. roggenkampii*, which harboured the *MIR* gene, all the other Enterobacter species possessed the *ACT* gene for AmpC. On the other hand, *K. aerogenes* did not carry the AMR gene encoding AmpC. The rates of nonsusceptibility to third-generation cephalosporins were 37.2% (*n* = 32) for ECC and 44.8% (*n* = 13) for *K. aerogenes*. Among the cases examined herein, third-generation cephalosporins were used as empirical or definitive therapy in two ECC BSI patients and three *K. aerogenes* BSI patients. However, no instances of acquired resistance leading to treatment failure or relapse were observed.

In a previous Chinese study of the AMR genes of 172 ECC isolates, a total of 80 resistance genes associated with 11 antimicrobial classes were detected across all the isolates. The most common resistance genes were related to β-lactams (41.4%), followed by aminoglycosides. Among the identified β-lactamase genes, the *ACT* gene was the most prevalent (58.7%), followed by the *TEM* gene (8.5%). Six carbapenemase-encoding genes (*NDM*-1, *NDM*-5, *IMP*-1, *IMP*-4, *IMP*-26, and *KPC*-2) were identified [21]. In the present study, *E. hormaechei* presented a relatively high prevalence of resistance genes. Among these genes, aminoglycoside resistance genes were the most dominant, with the *AAC* and *aadA* types being the most common and strongly correlated with phenotypic resistance. For β-lactam resistance, *LAP* and *SHV* genes were occasionally detected, whereas *TEM* genes were found at a relatively high frequency. Carbapenem resistance genes, as well as *CTX*-*M* and *OXA* types, were rarely identified. In contrast, resistance genes for TMP-SMX were detected in most *E. hormaechei* isolates. The *Qnr* gene, which mediates quinolone resistance, was less common, which aligns with the relatively low quinolone resistance rates. Overall, the ECC harboured more resistance genes than did *K. aerogenes* but showed an inconsistent relationship between major resistance genes and phenotypic resistance.

This study has several limitations. First, it was conducted within a single healthcare system, which may limit the generalizability of our findings to other settings. However, given the relatively large number of patients enrolled over a 14-year period, we believe that our results still provide meaningful insights. Additionally, this study focused solely on detecting the presence of resistance genes without assessing their expression levels and potential genetic mutations influencing antibiotic resistance. Nonetheless, since the presence of these genes is a fundamental prerequisite for antibiotic resistance mechanisms, our results provide a valuable foundation for understanding the resistance profiles and mechanisms of these bacterial strains. Finally, unlike traditional short-read sequencing, this study employed long-read sequencing for WGS. While long-read sequencing has a relatively high error rate, which may affect reliability, this limitation was addressed by using deep sequencing coverage. Gene presence was determined on the basis of sequence homology, ensuring a more robust and accurate assessment despite the inherent challenges of the technique.

In conclusion, this study provides valuable insights into the clinical and microbiological characteristics of *Enterobacter* spp. and *K. aerogenes*, highlighting their resistance patterns, genotypic diversity, and clinical outcomes in paediatric patients. These findings emphasize the need for continued surveillance of antimicrobial resistance, particularly increasing carbapenem resistance and the potential for AmpC-mediated inducible resistance. Additionally, the diverse genotypic landscape observed in both ECC and *K. aerogenes* suggests that clonal expansion of MDR strains has not yet become widespread, reinforcing the importance of effective antimicrobial stewardship and infection control measures. Moreover, species-level resolution within ECC improved interpretability by revealing differential AMR determinant profiles across ECC species.

## Figures and Tables

**Table 1:** Demographics of children with bloodstream infection caused by the *Enterobacter cloacae* complex and *Klebsiella aerogenes* between 2011 and 2024.

	No. of Cases (%)			*p*-Value *
	Total	ECC	*K. aerogenes*	
	(*n* = 115)	(*n* = 86)	(*n* = 29)	
Sex, male	54 (47.0)	43 (50.0)	11 (37.9)	0.286
Median age (IQR), years	4.3 (0.3–11.9)	7.8 (1.2–13.0)	0.3 (0.1–4.3)	0.001
Underlying disease				
Haemato-oncologic	58 (50.4)	52 (60.5)	6 (20.7)	<0.001
Preterm (GA < 34 weeks)	18 (15.7)	10 (11.6)	8 (27.6)	0.031
Gastrointestinal/hepatobiliary	18 (15.7)	14 (16.3)	4 (13.8)	1.000
Congenital heart disease	15 (13.0)	9 (10.5)	6 (20.7)	0.121
Diagnosis				
Urosepsis	8 (7.0)	3 (3.5)	5 (17.2)	0.027
CLABSI	67 (58.3)	52 (60.5)	15 (51.7)	0.360
Empirical antibiotics				
3rd-generation cephalosporin	7 (6.1)	2 (2.3)	5 (17.2)	0.012
Piperacillin–tazobactam	17 (14.8)	13 (15.1)	4 (13.8)	1.000
Cefepime	3 (2.6)	1 (1.2)	2 (6.9)	0.164
Meropenem	88 (76.5)	70 (81.4)	18 (62.1)	0.029
Antimicrobial susceptibility to				
empirical antibiotics used	107 (93.0)	80 (93.0)	27 (93.1)	1.000
Outcome				
Cure	104 (90.4)	79 (91.9)	25 (86.2)	0.471
Recurrence	6 (5.2)	5 (5.8)	1 (3.4)	1.000
Mortality, total in hospital	19 (16.5)	11 (12.8)	8 (27.6)	0.077
attributable	9 (7.8)	5 (5.8)	4 (13.8)	0.234
all-cause 30 d	11 (9.6)	5 (5.8)	6 (20.7)	0.022
all-cause 100 d	18 (15.7)	10 (11.6)	8 (27.6)	0.050

* Comparison between the ECC and *K. aerogenes* groups No, number; ECC, *Enterobacter cloacae* complex; IQR, interquartile range; GA, gestational age; CLABSI, central-line associated bloodstream infection; d, day.

**Table 2:** Prevalence of AMR genes in the *Enterobacter cloacae* complex and *Klebsiella aerogenes* strains.

Target Antibiotics and AMR Genes	*E. hormaechei* (*n* = 26)	Other ECC Strains (*n* = 11)	*K. aerogenes* (*n* = 9)
Aminoglycoside			
*AAC*	8 (30.8)	1 (9.1)	1 (11.1)
*aadA*	12 (46.2)	2 (18.2)	0 (0)
*ANT*	6 (23.1)	0 (0)	0 (0)
*APH*	7 (26.9)	3 (27.3)	1 (11.1)
Beta-lactam			
*ACT (ampC)*	26 (100)	9 (81.8)	0 (0)
*MIR (ampC)*	0 (0)	2 (18.2)	0 (0)
*CTX-M*	5 (19.2)	2 (18.2)	1 (11.1)
*LAP*	2 (7.7)	1 (9.1)	0 (0)
*OXA*	2 (7.7)	0 (0)	1 (11.1)
*SHV*	5 (19.2)	0 (0)	0 (0)
*TEM*	8 (30.8)	3 (27.3)	1 (11.1)
Bactrim			
*dfrA*	17 (65.4)	4 (36.4)	1 (11.1)
*sul*	21 (80.8)	4 (36.4)	1 (11.1)
Quinolone			
*Qnr*	10 (38.5)	3 (27.3)	1 (11.1)
Tetracycline			
*tet*	4 (15.4)	2 (18.2)	1 (11.1)
Colistin			
*MCR*	11 (42.3)	2 (18.2)	0 (0)
Antiseptics			
*qacE*	17 (65.4)	3 (27.3)	0 (0)

All data were presented as number (percentage). AMR, antimicrobial resistance; ECC, *Enterobacter cloacae* complex; *n*, number. ‘Other ECC’ includes *E. kobei* (*n* = 4), *E. asburiae* (*n* = 3), *E. ludwigii* (*n* = 2), *E. roggenkampii* (*n* = 2), and *E. bugandensis* (*n* = 1). *blaMIR* was detected in the *E. roggenkampii* isolates (*n* = 2), whereas *blaACT* was primarily observed in *E. hormaechei*.

## Data Availability

The data that support the findings of this study are openly available in 4TU.ResearchData at https://doi.org/10.4121/fe0f9938-0411-4aec-a39b-ecebf7ba8b30 (accessed on 22 January 2026).

## Supplementary Materials

The following supporting information can be downloaded at: https://www.mdpi.com/article/10.3390/microorganisms14020292/s1, Table S1: MinION sequencing quality metrics for the 47 isolates.

## Abbreviations

The following abbreviations are used in this manuscript:

GNBs	Gram-negative bacteria
ESBL	extended-spectrum β-lactamase
CRE	carbapenem-resistant Enterobacteriaceae
ECC	Enterobacter cloacae complex
BSI	bloodstream infection
SNUCH	Seoul National University Children’s Hospital
CDW	clinical data warehouse
AST	antimicrobial susceptibility testing
CLSI	Clinical and Laboratory Standards Institute
IRB	institutional review board
WGS	whole-genome sequencing
MLST	multilocus sequence typing
ST	sequence type
IQR	interquartile range
HOD	haemato-oncologic disease
CLABSI	central line-associated BSI
PIP-TAZ	piperacillin–tazobactam
TMP-SMX	trimethoprim–sulfamethoxazole
OR	odds ratio
CI	confidence interval
