# Supplementary material for: Clinical and Genetic Characteristics of Enterobacter cloacae and Klebsiella aerogenes in Children
Source: Microorganisms. 2026 Jan 27;14(2):292. doi: 10.3390/microorganisms14020292 (PMC12943620; doi:10.3390/microorganisms14020292)
Supplement: Supplementary file 1 [file microorganisms-14-00292-s001.zip › microorganisms-4052431-supplementary.pdf]

Table S1. MinION sequencing quality metrics for the 47 isolates.

Strain	Species	Contigs (n)	Max (Kbp)	Average (Kbp)	N50 (K)	Coverage Depth (X)
11-252	*E. hormaechei*	4	2534	1187	2534	64.5
11-285	*E. hormaechei*	3	5064	1801	5064	98.5
11-299	*E. hormaechei*	3	4761	1763	4761	57.4
11-404	*E. hormaechei*	4	4694	1215	4694	53.0
11-430	*E. hormaechei*	7	5023	771	5023	206.3
12-119	*K. aerogenes*	3	4554	1765	4554	134.8
12-288	*E. hormaechei*	3	4087	1714	4087	97.8
13-146	*E. ludwigii*	2	4911	2589	4911	29.9
13-257	*E. hormaechei*	4	4707	1277	4707	62.7
14-023	*E. hormaechei*	1	4859	4859	4859	80.6
14-144	*K. aerogenes*	4	5128	1344	5128	396.4
14-228	*K. aerogenes*	2	5213	2724	5213	218.0
15-043	*E. asburiae*	8	4842	679	4842	47.1
15-214	*E. hormaechei*	4	4659	1222	4659	210.2
15-267	*E. hormaechei*	3	4732	1690	4732	205.6
16-269	*E. kobei*	4	4711	1217	4711	58.3
16-281	*E. hormaechei*	3	4749	1690	4749	58.7
16-456	*E. hormaechei*	5	5104	1076	5104	266.0
17-143	*E. hormaechei*	2	4586	2432	4586	113.5
17-189	*E. asburiae*	5	4712	979	4712	96.6
17-262	*E. hormaechei*	1	4804	4804	4804	173.7
17-263	*E. bugandensis*	2	4715	2402	4715	377.5
18-058	*E. hormaechei*	4	4897	1307	4897	280.1
18-199	*K. aerogenes*	2	5295	2680	5295	57.8
18-256	*E. hormaechei*	3	4580	1639	4580	34.8
19-020	*E. roggenkampii*	3	4803	1638	4803	287.9
19-038	*E. kobei*	3	4691	1581	4691	58.1
19-332	*E. hormaechei*	3	4660	1574	4660	96.7
20-102	*K. aerogenes*	2	5353	2681	5353	90.7
20-166	*E. hormaechei*	2	4829	2420	4829	161.3
21-110	*K. aerogenes*	1	5313	5313	5313	84.9
21-136	*E. asburiae*	3	4643	1611	4643	98.2
21-220	*E. hormaechei*	10	1219	474	1193	16.9
21-223	*E. kobei*	4	4711	1217	4711	58.3
21-246	*E. roggenkampii*	6	4932	838	4932	137.2
22-005	*E. ludwigii*	3	3546	1632	3546	17.6
22-022	*E. hormaechei*	2	5077	2613	5077	62.5
22-054	*E. hormaechei*	3	4762	1639	4762	121.8
22-071	*E. hormaechei*	7	4759	717	4759	152.5
22-077	*K. aerogenes*	2	5272	2641	5272	112.1
22-121	*E. hormaechei*	2	5079	2614	5079	154.4
22-139	*E. hormaechei*	3	4634	1644	4634	231.7
23-175	*E. hormaechei*	3	4773	1646	4773	107.2
23-177	*K. aerogenes*	1	5162	5162	5162	340.1
23-256	*E. hormaechei*	2	4699	2416	4699	362.3
24-070	*E. kobei*	6	2894	805	2894	166.5
24-087	*K. aerogenes*	2	5279	2644	5279	27.7
